# Tuning Fluidic Resistance via Liquid Crystal Microfluidics

**DOI:** 10.3390/ijms141122826

**Published:** 2013-11-19

**Authors:** Anupam Sengupta

**Affiliations:** Max Planck Institute for Dynamics and Self Organization (MPIDS), Am Faßberg 17, 37077 Göttingen, Germany; E-Mail: anupam.sengupta@ds.mpg.de; Tel.: +49-551-5176-214; Fax: +49-551-5176-202

**Keywords:** microfluidics, liquid crystals, surface anchoring, flow resistance, flow circuits

## Abstract

Flow of molecularly ordered fluids, like liquid crystals, is inherently coupled with the average local orientation of the molecules, or the director. The anisotropic coupling—typically absent in isotropic fluids—bestows unique functionalities to the flowing matrix. In this work, we harness this anisotropy to pattern different pathways to tunable fluidic resistance within microfluidic devices. We use a nematic liquid crystalline material flowing in microchannels to demonstrate passive and active modulation of the flow resistance. While appropriate surface anchoring conditions—which imprint distinct fluidic resistances within microchannels under similar hydrodynamic parameters—act as passive cues, an external field, e.g., temperature, is used to actively modulate the flow resistance in the microfluidic device. We apply this simple concept to fabricate basic fluidic circuits, which can be hierarchically extended to create complex resistance networks, without any additional design or morphological patterning of the microchannels.

## 1. Introduction

The advent of microfluidics has opened up possibilities to study flows within micron-sized confinements and past minute obstacles. It has evolved as the major technological breakthrough for development of interdisciplinary research—converging physics, chemistry, biology, and technology—all on one platform [1]. In contrast to the flows at macro scales, flow within microchannels is fundamentally different due to the drastic reduction of the inertial effects, resulting in very low Reynolds numbers [2]. Microfluidics derives its forte from the ability to control and manipulate flows precisely. Using a range of available techniques [3], the conduits can be fabricated with high dimensional precision for manipulating flow: micro-pumps, micro-valves, and flow-guiding paths [4]. Such individual components can be subsequently put together to construct large-scale-integrated fluidic networks of varied levels of complexity, resulting in highly efficient lab-on-a-chip devices [5]. In general, the use of complex fluids as the transport medium in microfluidic confinements has provided interesting pathways to create and study novel phenomena, leading to numerous applications. For instance, the use of emulsions, where typically aqueous droplets are embedded in an immiscible carrier fluid such as oil, has become known as droplet-based microfluidics [6]. More recently, topological microfluidics—an emerging area in microfluidics research and application—harnesses complex anisotropic interactions between the molecular structure of liquid crystalline phases with the geometrical constraints provided by the microchannels, creating a versatile playground for a range of novel phenomena, and potentially, novel applications on microfluidic platforms [7].

Liquid crystal (LC) materials, since their discovery in the late 19th century, have been widely studied for their interesting, and at times unique, anisotropic properties. In the context of fluid flows, LCs generally show complex non-Newtonian behavior. The average orientation of the molecules, commonly called the director, *↛*, plays a decisive role in determining the rheological properties of the anisotropic liquid crystalline phases. This was, for the first time, reported by M. Miesowicz in 1946, when the existence of three different viscosity coefficients in anisotropic liquids was experimentally demonstrated [8]. The apparent viscosity of the system was lowest when the director—held fixed using a strong magnetic field—oriented parallel to the flow, and highest when it was perpendicular. The third, and the intermediate value was observed when the director was perpendicular to both the flow and its gradient. As a consequence of the three viscosity coefficients, and the inherent coupling between the flow and the director fields [9,10], the dynamics of the LC flows are rich in complexity, leading to a variety of interesting phenomena like the generation of a transverse pressure gradient in a Poiseuille flow [11], anomalous colloidal rheology [12], and tunable flow shaping in microfluidic confinements [13].

In this paper, we present a simple concept of tuning fluidic resistance in microfluidic channels by utilizing the anisotropic flow-director coupling inherent in liquid crystalline materials. For a given microchannel, the hydrodynamic resistance experienced by a flow generally scales with the dynamic viscosity of the flowing matrix. Alternatively, for a given fluidic material, the hydrodynamic resistance can be modulated by altering the channel dimensions [14], architecture [15], or by changing the composition of the flow matrix [16]. Here we employ appropriate boundary conditions, *i.e.*, surface-induced anchoring of a nematic liquid crystal (NLC) material on the channel walls, to create tunable fluidic resistance. By modifying the equilibrium orientation of the NLC molecules in the static case, different flow speeds, *v*, and equivalently different fluidic resistances, *R_flow_*, were obtained under comparable pressure gradients within microchannels of similar dimensions. Distinct fluidic resistances were then imprinted at different parts of a microchannel using available surface functionalization techniques. The flow of the liquid crystal matrix through such channels could be guided preferentially along the low resistance pathways. Furthermore, we use an external field, e.g., temperature, to actively tune the flow resistance within the channels. The concepts demonstrated here can be applied to fabricate microfluidic networks of higher complexity for guiding and patterning microflows using anisotropic fluids.

## 2. Results and Discussion

### 2.1. Estimation of the Dynamic Viscosities

Estimation of the dynamic viscosity constitutes the first step towards characterizing the fluidic resistance of nematofluidic systems. The dynamic viscosities were measured under different boundary conditions (surface anchoring) over a range of driving pressures applied along the channel length. The inlet of the microchannel was connected to a pressure controller (discussed in Section 2.2), and the outlet was left at atmospheric pressure. For ensuring a reduced influence of the side walls, high aspect ratio (width/depth = *w*/*d* ~ 133) microchannels were considered for the viscosity measurements. The width, *w* was maintained at ~2 mm, whereas the channel depth, *d*, was fixed at ~15 μm. The nematic director in absence of any flow was determined by the surface-induced anchoring within the microchannels. Based on this initial director equilibrium with respect to the flow direction, *v⃗*, two limiting cases of the flow-director interactions emerge:

a) homeotropic anchoring: *↛* ⊥ *v⃗* and ∇*v⃗* || *↛*, and
b) uniform planar anchoring: *↛* || *v⃗*.

**Figure 1:**
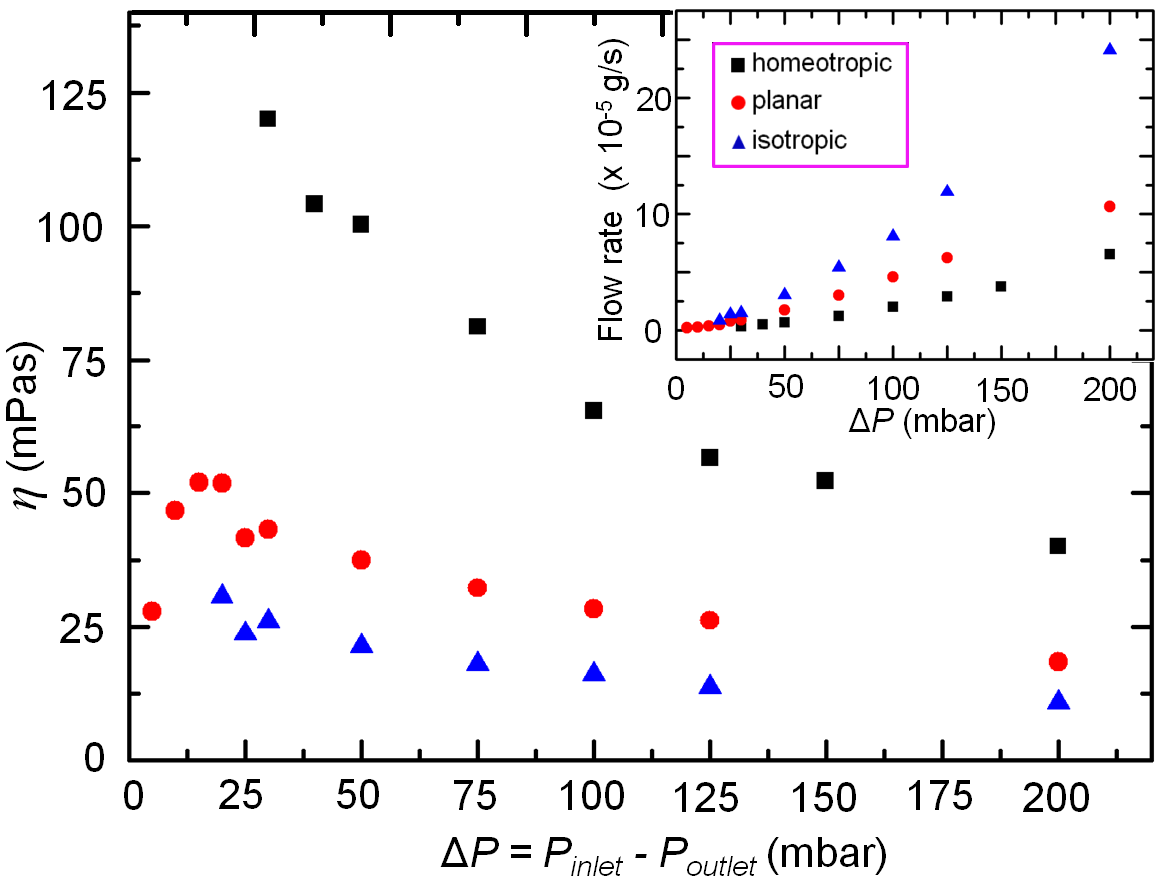
Effective viscosity of 5CB, *η*, measured experimentally using pressure-driven flow through microchannels possessing homeotropic (black squares), and uniform planar (red circles) surface anchoring. The effective viscosity for the flow in isotropic phase is shown by the blue triangles. The measurements for the homeotropic and the uniform planar anchoring cases were done at room temperature. The inset plots the measured mass flow rate for the three cases as a function of applied pressure difference.

Additionally, measurements were carried out for 5CB in isotropic phase (~35 °C). Figure 1 plots the experimental estimation of the effective dynamic viscosity of 5CB as a function of the applied pressure difference Δ*P = P_inlet_− P_outlet_*. The effective viscosity, η, was calculated by measuring the mass flow rate of 5CB at different pressure differences from the well-known relation:

(1) $$\begin{array}{l} \frac{\Delta P}{l}=\frac{A\eta }{wd^{3}}\left(\frac{\dot{m}}{\rho }\right)\\ \eta =\frac{wd^{3}}{A}\left(\frac{\Delta P}{l}\right)\frac{\rho }{\dot{m}} \end{array}$$

Here, *w*, *d*, and *l* ~ 4 cm denote the channel dimensions. ρ, Δ*P*, and *ṁ* signify 5CB density, applied pressure difference, and the measured mass flow rate, respectively. Two different values of 5CB density were considered: in nematic phase ρ = 1025 kg/m^3^, and in isotropic phase ρ = 1013 kg/m^3^[17]. The prefactor *A* in Equation 1 depends upon the geometric dimensions of the channel [16,18], and was evaluated from the experiments: The flow rate of NLC 5CB was measured at a pressure difference of 200 mbar within a channel having no specific anchoring (degenerate anchoring) conditions. Using the average bulk viscosity of 5CB ~30 mPas at room temperature, the value of *A* = 1.48 was experimentally determined for the given dimensions of the microchannel. The expansion of PDMS (either due to high pressures or due to elevated temperatures), and the pressure drop at the inlet tubing of the channel (~0.3 mbar at maximum flow rates), were neglected in determining the actual pressure at the inlet port. As is evident from Equation 1, the relative change of the effective viscosity is affected only by the change in the mass flow rate, for a given microchannel under similar thermodynamic conditions.

The dynamic viscosity of the system was estimated from Equation (1). For each value of the applied pressure, the mass of the NLC 5CB flowing out of the channel was measured over time. By varying the pressure at the channel inlet, we obtained the dependence of the mass flow rate on the pressure difference along the channel. Measurements were carried out for homeotropic, uniform planar and isotropic flow cases, and plotted in Figure 1 (see inset). The effective viscosity for each of the cases is presented in Figure 1. The measured viscosities of 5CB clearly varied with the magnitude of the driving pressure, and additionally, on the nature of the channel boundary conditions. At low driving pressures, the dynamic viscosity was observed to be significantly higher (up to ~5 times) for homeotropic conditions (*η*_H_), compared to the flow where the velocity and initial director were mutually parallel (*η*_P_). The measurements of 5CB flow in isotropic phase indicated lowest effective viscosity for the entire range of applied pressure difference. Consequently, for a similar difference of pressure between the inlet and the outlet ports, nematic 5CB flowed with the slowest speed in the homeotropic channel, whereas the flow in isotropic phase was the fastest. The difference between the effective viscosities of the homeotropic and uniform planar cases is particularly remarkable at applied pressures Δ*P* < 100 mbar (Figure 1): as the pressure increases the relative difference drops. This can be understood by considering the competing effects of the flow-induced viscous torque, and the surface-induced elastic torque, characterized by the non-dimensional number, Ericksen number, *Er*. Typically, *Er* >> 1 suggests a dominating influence of the viscous forces over its elastic counterpart. In the present experiment, this value varied between 0 < *Er* < 250 for the homeotropic, and 0 < *Er* < 160 for the uniform planar flows. At low driving pressures, and hence low viscous torques, the flow-induced distortion of the initial homeotropic alignment is marginal due to a dominating effect of the counter elastic torque from the surface-aligned director. Correspondingly, the anisotropic coupling between the flow and the nematic viscosity (which depends on the local director orientation) leads to a reduced transport of the nematic matrix. In contrast, flow under uniform planar conditions is more favorable, since the director orientation offers a minimum counter elastic torque. As the applied pressure is increased for the homeotropic channel, the viscous torque begins to overcome the elastic torque, and the director of the nematic 5CB aligns gradually along the flow direction. This results in an easier transport of the nematic matrix, and resulting in a lower effective viscosity [13]. At high enough pressure differences in a homeotropic channel, the molecules are aligned almost along the flow direction, the effective viscosity thereby approaching values similar to that of the uniform planar case (see Figure 1). Additionally, with increase in the applied pressure, the absolute value of the effective viscosity experiences a steeper drop for the homeotropic case (~125 mPas to 50 mPas), in contrast to the uniform planar case (~50 mPas to 18 mPas). Experiments carried out in hybrid channels with anchoring on the glass surface parallel to the flow direction (|| planar, see Figure 8b) yielded effective viscosities similar to those of *η*_P_ ≈ *η*_||_ over the range of pressure difference considered here. The experimental values for *η*_⊥_ (hybrid _⊥_ planar) were found to lie between *η*_H_ and *η*_P_ Interestingly, the variation of the viscosity within the planar microchannel shows an initial rise, before going down at higher applied pressures. This variation of *η*_P_ at low inlet pressures (Δ*P* ~ 25 mbar) can be attributed to the flow-induced director reorientation of the planar aligned molecules [19]. Due to the interplay of the viscous and the elastic torques, the director reorients slightly away from the initial state (*↛* || *v⃗* ) on starting the flow. Consequently, the effective viscosity has an additional contribution from the projection of the director component out of the plane containing the flow and director fields. The reorientation continues till the viscous torque vanishes at the critical Leslie angle [19]. Thereafter, the viscosity reduces monotonically, as in the case of the homeotropic boundary conditions. By varying the applied pressure difference by ~200 mbar along the given microchannel, we could tune the relative change in the dynamic viscosity over the range 2 < *η*_H_/*η*_P_ < 3.5. The overall behavior of the nematic microflow is in accordance to the expected shear thinning trend of the bulk 5CB [20]. The present experiments additionally show that the shear thinning nature of 5CB holds true for each anchoring case, and also for the isotropic phase.

**Figure 2:**
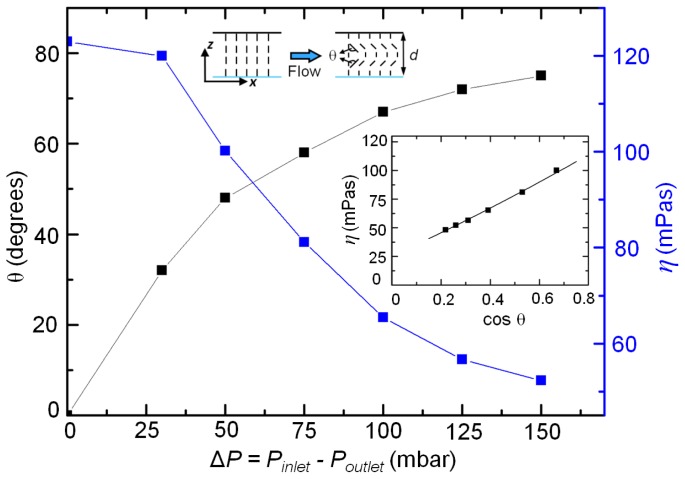
Average distortion of the nematic director, θ (inset schematic), plotted as a function of the applied pressure within microchannels with homeotropic surface anchoring. The corresponding variation of the effective viscosity is shown in blue. The inset graph plots the measured viscosity as a function of the director distortion.

The variation of the dynamic viscosity is particularly prominent within microchannels having homeotropic anchoring. With increasing driving pressure, the nematic director distorts relative to the initial orientation. The average distortion of the nematic director, θ (see inset schematic, Figure 2), was experimentally estimated from the optical birefringence measurements [21]. We have used polarization optical microscopy to record the flow-induced birefringence (and its order). Within a microchannel of known depth, the birefringence was then used to calculate the average director distortion. As plotted in Figure 2, the distortion angle θ (black squares) exhibits a non-linear rise with increasing driving pressure (and hence, flow speed), shown here for a 15 μm deep channel. The blue squares plot the corresponding decrease in the effective viscosity. The dependence of the nematic viscosity at any general value of the director distortion was obtained by plotting the former as a function of cosθ = || *v⃗*×*↛*||/(||*v⃗*|| ||*↛*||) [22]. The inset plot in Figure 2 presents the variation of the effective viscosity with cos θ, which was fitted using a second degree polynomial as:

(2) $$\eta_{\theta }=p_{1}\;{\text{cos}}^{2}\;\theta +p_{2}\;\text{cos }\theta +p_{3}$$

The values of the coefficients were found to be *p*_1_ = 15.3, *p*_2_ = 98.3, and *p*_3_ = 25.4, all in the units of mPas. In the present configuration of the flow and director fields, the nematic director cannot withstand viscous torques, and consequently deform even with small driving pressures. Using the above relation, the effective viscosity in the limit of no flow situation (Δ*P* → 0, θ → 0), is evaluated to be 139 mPas. This is within 15% of the reported 5CB viscosity, measured under homeotropic (*↛* ⊥ *v⃗* and ∇*v⃗* || *↛* ) condition [17]. In the limit of flow alignment of the nematic director, θ → θ*_Leslie_* ~78 degrees [21], the effective viscosity is estimated to be 46 mPas. Thus, over the entire range of the angular distortion, the apparent viscosity drops by ~90 mPas in a homeotropic microchannel. The trend observed in our experiments agrees well that calculated using a friction model for shear flows of NLCs [22]. However, the rate at which the viscosity drops (with the director deformation) is observed to be slower in our experiments, as is evident from the slope of the fitting function.

### 2.2. Estimation of the Fluidic Resistance

The fluidic resistance in a microchannel bears direct analogies with the electrical resistance of a current carrying wire [18]. In the simplest case of an electrical circuit, the resistance is measured as the ratio $R_{elec}=\frac{\Delta V}{I}$, where *R_elec_*, Δ*V*, and *I* refer to the electrical resistance, applied voltage, and the current flow respectively. The electrical resistance, *R_elec_*, takes into account the conductor geometry and the material resistivity, the latter obtained from the Drude model of electron scattering [23].

In the parlance of microfluidics, the relevant transport occurs due to the driving pressure across the channel. Hence, the fluidic resistance in a channel, *R_flow_*, is obtained as the ratio between the driving pressure difference and the volume flow rate:

(3) $$R_{flow}=\frac{\Delta P}{Q}=\frac{Al}{wd^{3}}\eta$$

where, Δ*P* is the applied pressure difference (analogous to the applied voltage, Δ*V* ), and $Q=\frac{\dot{m}}{\rho }$ is the volume flow rate (analogous to the current flowing in an electrical circuit, *I*). In analogy to the electrical resistance, here $\frac{Al}{wd^{3}}$ is dependent on the channel geometry, and *η* acts as the material resistivity. Correspondingly, the fluidic resistance per unit length of the channel is:

(4) $$r_{flow}=\frac{R_{flow}}{l}=\frac{\Delta P/l}{Q}$$

which, using Equation 1 reduces to:

(5) $$r_{flow}=\left(\frac{\Delta P/l}{\dot{m}/\rho }\right)=\frac{A}{wd^{3}}\eta$$

The analysis of fluid flows as electrical circuits allows us to apply equivalent rules to fluidic networks. For instance, the well known Kirchhoff’s current and voltage laws can be used to estimate the flow rates and pressure drops across different elements of a fluidic network. In case of nematofluidics, one has to take into account the apparent flow-induced viscosity, depending upon the existing boundary conditions. Consequently, the alignment of the nematic director relative to the flow (which can be tuned by the nature of surface anchoring) additionally influences the effective fluidic resistance in the nematofluidic circuits. Corresponding to the measurements in Figure 1, the fluidic resistance per unit length of the homeotropic, and planar channels varied from 8.4 < *r_H_* < 27, and 4 < *r_P_* < 11 respectively, while it ranged over 2.5 < *r_iso_* < 6.3 for the flow in isotropic phase. The values are in the units of 10^15^ Pas/m^4^.

#### Influence of the Channel Depth

As derived in Equation (5), the fluidic resistance in a channel scales with the channel dimensions as $r_{flow}\propto \frac{A}{wd^{3}}$. For an infinitely wide channel, the value of *A* approaches a numerical constant. Thus, for a channel transporting a Newtonian fluid (constant *η*), the hydrodynamic resistance is influenced due to the channel geometry only: for instance, *d* → *d*/2, *r_flow_* → 8*r_flow_*. However, in case of nematic microflows, the effective enhancement of the resistance should have an additional component arising due to the coupling between the flow and the director fields (*η* = *f*(ν)). Precisely, one has to take into account not only the effect of the flow speed, but also of the confinement, on the apparent viscosity term. Due to the large variation of the effective viscosity over a given range of applied pressure (see Figure 2), homeotropic microchannels have been considered here for evaluating the influence of the channel depth.

**Figure 3:**
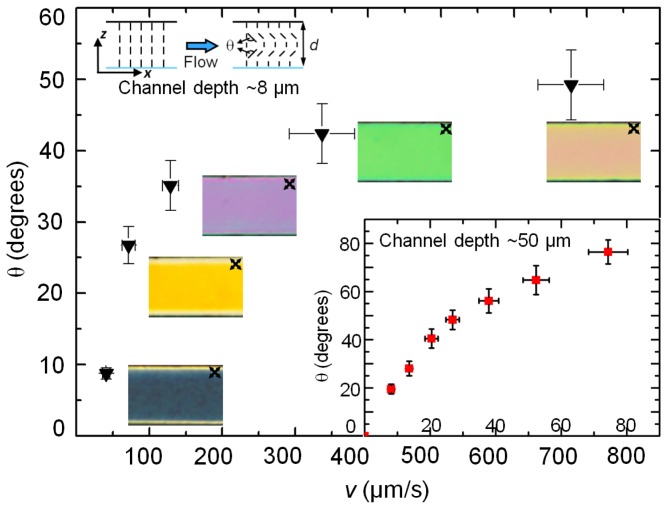
Reorientation of the nematic director field due to flow-induced viscous stress. The angle of reorientation, θ (shown in the schematic), is plotted as a function of the flow speed within a ~8 μm deep channel. The reorientation was measured by quantifying the birefringence observed at different flow speeds. The corresponding birefringence in the microchannel is shown here using polarization optical micrographs (the crossed-arrows indicate the orientation of the polarizers). The inset graph presents the angle of reorientation within a ~50 μm deep channel. The channel width in both cases was 100 μm.

Figure 3 plots the flow-induced deformation of the nematic director in two different channels with depths, *d* ~ 8 μm and 50 μm, both having width, *w* = 100 μm. The flow speed was measured by tracking tracer particles, whereas the director distortion was estimated by quantifying the flow-induced birefringence within the homeotropic channels. From the initial homeotropic alignment, the nematic director begins to rotate along the flow direction. At low enough driving pressures, the viscous torque is countered by the surface-induced elastic torque, thereby hindering the director deformation. With increasing driving pressure, the average deformation angle, θ, increases until a saturation value (~78 degrees for 5CB at room temperature [21]) is reached at which the viscous torque on the director vanishes. In Figures 1 and 2, we have presented the corresponding reduction of the apparent viscosity.

The perturbation of the director field can be visually observed in white light microscopy by placing the sample between crossed polarizers. Because of the homeotropic anchoring conditions, the sample exhibits, at zero flow, apparently no birefringence along the *z* direction. On increasing the flow speed *v* (and thus increasing θ), the effective birefringence along *z* increases and, for an appropriate sample thickness, one can expect a sequence of interference colors to appear [21]. Figure 3 shows the polarized micrographs of the series of interference colors corresponding to different flow speeds as observed in an 8 μm deep microchannel. The director deformed over 7 < θ < 49 degrees as the flow speed was varied between ~10 to 700 μm/s. In contrast, the nematic director within the 50 μm deep channel attained the saturation angle at a significantly lower flow speed, *v* ~ 80 μm/s (see Figure 3, inset). It is thus easier to distort the static director field within a deeper channel – due to a weaker surface-induced elastic interaction—as compared to a shallow one, in which the strong confinement conditions assist to stabilize the homeotropic director orientation. For instance, to distort the director by ~40 degrees, one requires a flow speed of ~300 μm/s in a 8 μm deep channel, compared to ~20 μm/s in the 50 μm deep channel. Equivalently, as the characteristic length of the system (typically the channel depth) increases, the viscous torque overcomes the elastic torque at relatively lower flow speeds (and hence applied pressure difference). By introducing *η*_θ_ (from Equation (2)) in Equation (5), we can generalize the expression for the flow resistance:

(6) $$r_{flow}=\frac{A}{wd^{3}}\eta =\frac{A}{wd^{3}}\left(p_{1}\;{\text{cos}}^{2}\;\theta +p_{2}\;\text{cos }\theta +p_{3}\right)$$

At a given flow speed, the ratio of the fluidic resistances in the homeotropic channels of similar widths but with different channel depths, *d*_1_ > *d*_2_ is thus given by:

(7) $$\begin{array}{l} \frac{r_{H,1}}{r_{H,2}}=\frac{A_{1}}{w{d_{1}}^{3}}\left(p_{1}\;{\text{cos}}^{2}\;\theta_{1}+p_{2}\;\text{cos }\theta_{1}+p_{3}\right)/\frac{A_{2}}{w{d_{2}}^{3}}\left(p_{1}\;{\text{cos}}^{2}\;\theta_{2}+p_{2}\;\text{cos }\theta_{2}+p_{3}\right)\\ =\left[\frac{A_{1}}{A_{2}}{\left(\frac{d_{2}}{d_{1}}\right)}^{3}\right]\left(\frac{p_{1}\;{\text{cos}}^{2}\;\theta_{1}+p_{2}\;\text{cos }\theta_{1}+p_{3}}{p_{1}\;{\text{cos}}^{2}\;\theta_{2}+p_{2}\;\text{cos }\theta_{2}+p_{3}}\right) \end{array}$$

The first term in Equation (7), $\frac{A_{1}}{A_{2}}{\left(\frac{d_{2}}{d_{1}}\right)}^{3}={\bar{r}}_{hydrodyn}$ signifies the relative change in the fluidic resistance due to the channel geometry, whereas the second term, $\left(\frac{p_{1}\;{\text{cos}}^{2}\;\theta_{1}+p_{2}\;\text{cos }\theta_{1}+p_{3}}{p_{1}\;{\text{cos}}^{2}\;\theta_{2}+p_{2}\;\text{cos }\theta_{2}+p_{3}}\right)={\bar{r}}_{director}$, arises solely due to the average deformation of the director. Since, *d*_1_ > *d*_2_, for a given flow speed (or applied pressure difference), θ_1_ > θ_2_. Using the measured values of the director deformation from Figure 3: corresponding to *v* ~ 40 μm/s, θ_1_ ~ 56 degrees for *d*_1_ = 50 μm, and θ_2_ ~9 degrees for *d*_1_ = 8 μm, the enhanced fluidic resistance of the shallower channel is $r_{H,2}=\frac{r_{H,1}}{{\bar{r}}_{director}{\bar{r}}_{hydrodyn}}=1.61\left(\frac{r_{H,1}}{{\bar{r}}_{hydrodyn}}\right)$. This is about 60% more than that due to the usual hydrodynamic considerations.

### 2.3. Capillary Filling

**Figure 4:**
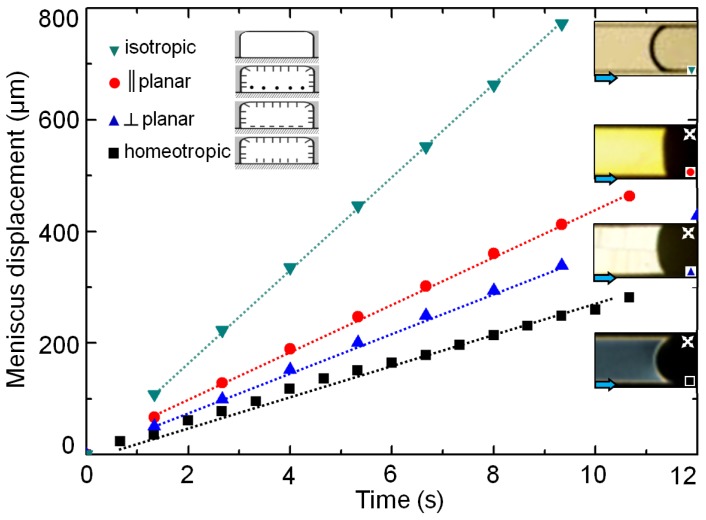
Time-displacement plots tracking the motion of the 5CB meniscus due to capillary action. Experiments were carried out at isotropic phase (~35 °C, inverted triangles in teal), and in nematic phase under three different surface anchoring conditions: hybrid || planar (red circles), hybrid ⊥ planar (blue triangles), and homeotropic (black squares). The dotted lines are for guiding purpose only. The corresponding *xy* profiles of the 5CB menisci are captured using polarization optical micrographs (the crossed-arrows indicate the orientation of the polarizers).

The anisotropic coupling between the flow and the director fields manifests itself most prominently at low flow speeds and small channel depths. Here we present a complementary characterization of this coupling, carried out by filling up the microchannels via capillary action. For the measurements, we have used 2 cm long microchannels of depth, *d* ~ 8 μm, and width, *w* = 100 μm, possessing three different surface anchoring conditions: homeotropic, hybrid || planar, and hybrid ⊥ planar (see Figure 4). A hole ~1 mm in diameter was punched on the PDMS relief at the entrance of the channels. The hole was filled with ~100 μL of nematic 5CB, which gradually flowed within the channels. The volume of the dispensed 5CB at the channel entrance was maintained sufficiently low to ensure a minimum gravity effect during the capillary filling process.

Figure 4 plots the displacement of the 5CB meniscus over time for different anchoring and thermodynamic conditions. The corresponding polarization optical micrographs (except for the isotropic case, which was visualized in unpolarized white light) of the menisci are presented as insets in Figure 4. The meniscus velocities were obtained by calculating the slopes of the displacement-time plots. While the isotropic front had the fastest displacement rate of ~82 μm/s, the meniscus of the homeotropic channel travelled with the minimum speed of ~25 μm/s. The speeds of the hybrid || planar, and hybrid ⊥ planar menisci were intermediate with *v*_||_ ~ 35 μm/s > *v*⊥ ~30 μm/s. The difference in the flow speeds between distinct anchoring conditions is crucial for flow selectivity, and will be subsequently employed for directing the nematic flow within a specific channel in a microfluidic network.

### 2.4. Transition to Isotropic Phase

**Figure 5:**
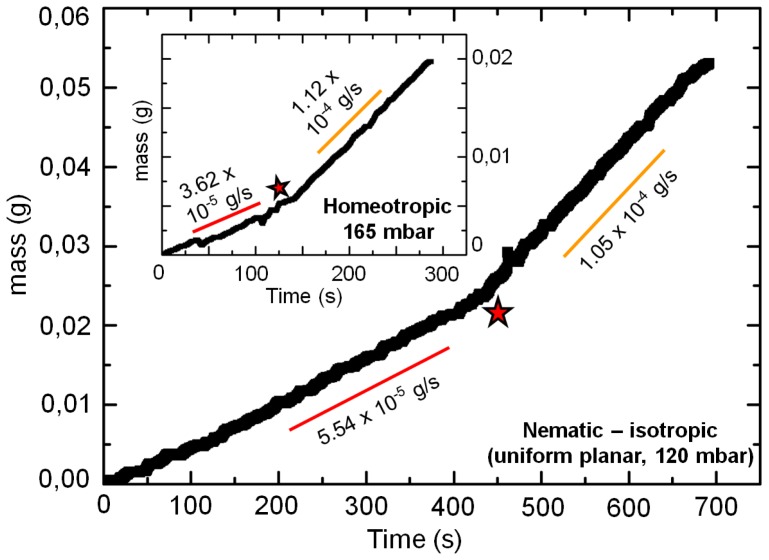
Pressure-driven flow before and after the nematic-to-isotropic phase transition, indicated by the star symbol. The mass flow rate increased by ~100 in the planar case (at 120 mbar), and by ~200% in the homeotropic case (165 mbar).

In Section 3.1, we have observed that 5CB in isotropic phase had the least effective viscosity over the entire range of applied pressure difference. In one of our previous works, we have demonstrated that the profile of a nematic microflow could be steered by applying a temperature gradient (between 25 °C and 32 °C) across the width of the channel [13]. Here, we carry out the experiments at an elevated temperature (~35 °C), so that the flowing nematic undergoes a transition to isotropic phase. The variation of the mass flow is plotted over time, as shown in Figure 5. The measured mass flow, acquired from microchannels possessing two distinct anchoring conditions—uniform planar and homeotropic—both indicate an enhancement after transition to isotropic phase. At a given value of applied pressure, the mass flow rate enhanced by ~100% in planar case (at 120 mbar), and by ~200% in homeotropic case (165 mbar) upon transition from the nematic to the isotropic phase. Correspondingly, the effective viscosity approximately reduced from 25 mPas to 13 mPas in planar case, and from 53 mPas to 17 mPas in the homeotropic confinement. The temperature-induced reduction of the effective viscosity in the liquid crystalline material is significant considering that a usual isotropic fluid undergoes only a marginal drop for the corresponding temperature variation [2].

The quantitative distinction, at the phase transition, between the two cases described above can be understood by analyzing the temperature-induced director alignment of 5CB. It is well established that the nematic liquid crystal molecules undergo thermal reorientation on variation of the temperature [24]. For instance, with increasing temperature, the NLC molecules of a homeotropic sample gradually reorient parallel to the confining surfaces. This can be readily visualized using polarization optical microscopy when an LC cell, filled with 5CB, is gradually heated up to the nematic-isotropic phase transition temperature. In the present context, such an occurrence reduces the effective viscosity within the homeotropic confinement more drastically, in comparison to the uniform planar case. The temperature dependence of flow-alignment in NLCs, close to the transition temperature, was investigated experimentally by Gähwiller [25], and the phenomenological relations were given by W. Helfrich [26,27]. The temperature-induced enhancement of the mass flow rate suggests a change in the flow profile. This is currently being investigated. On lowering the temperature, the flow-aligned nematic state is recovered. The degree of molecular order becomes larger again, and the effective viscosity is once again determined only by the relative orientation between the director and the flow fields.

### 2.5. Microfluidic Circuits via Composite Anchoring Templates

The characterizations of the nematic microflows presented here elucidate different possibilities to modulate fluidic resistance: surface anchoring, confinement, and temperature. In addition, electric field effects offer a promising approach to tune nematic flows [28,29]. The anisotropic coupling between the viscous and elastic interactions in a flowing NLC material is fundamental in modulating the flow properties. This is in contrast to isotropic fluids, wherein any variation is achieved generally through morphological patterning of the microfluidic architecture.

**Figure 6:**
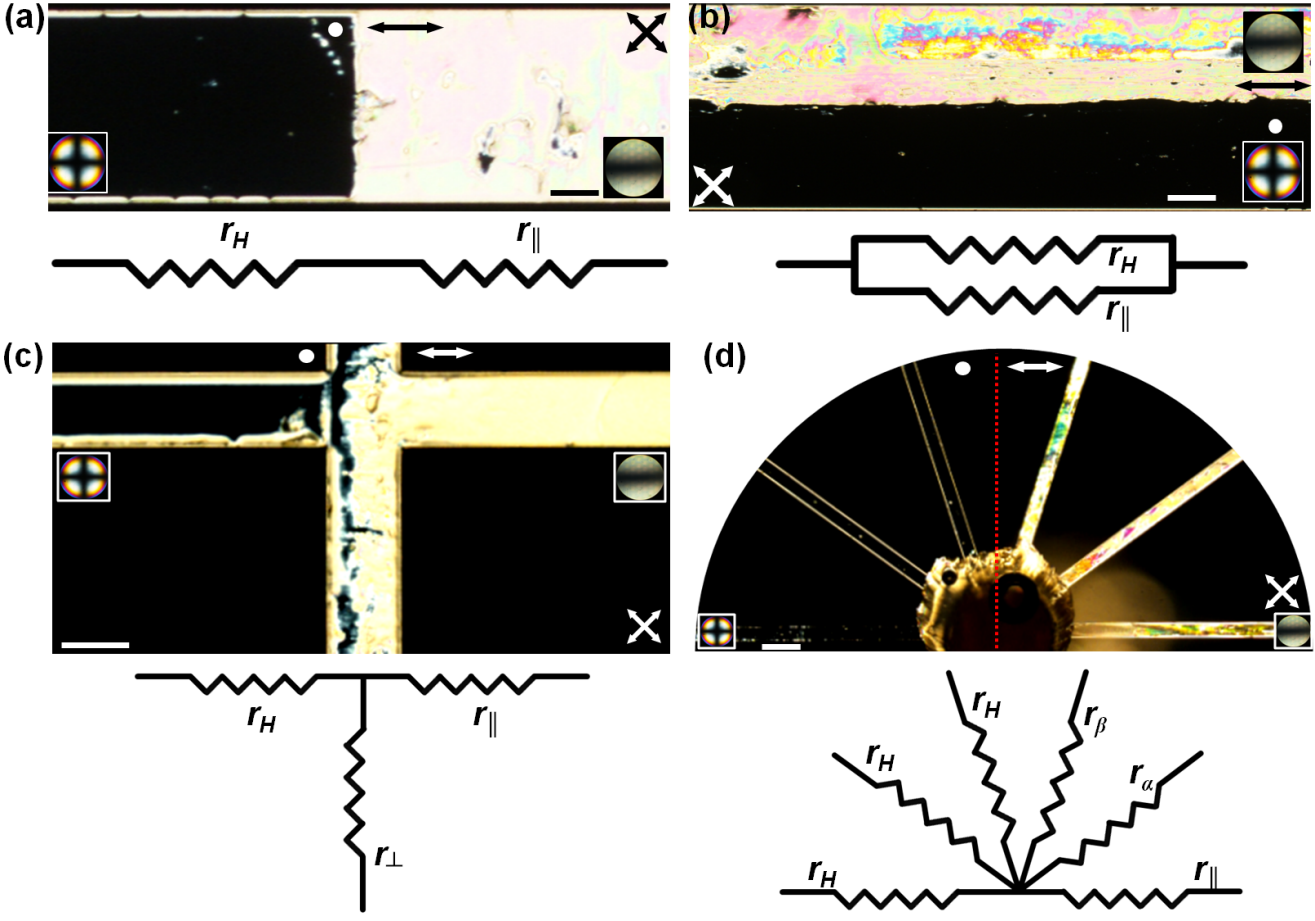
Resistance templates for simple microfluidic circuits. Polarization optical micrographs (the crossed-arrows indicate the orientation of the polarizers), and accompanying conoscopic images characterize the surface properties of the microchannels filled with nematic 5CB. The double-headed arrow, and the circular dot on each micrograph distinguish the hybrid || planar region from the homeotropic one. (**a**) two distinct fluidic resistances imprinted within the channel, each extending over *l*/2. The left half is homeotropic, *i.e.*, high resistance, *r_H_*, and the right half is hybrid || planar, *r*_||_, with a lower resistance. The conoscopic images at the bottom corners of the micrographs confirm the surface anchoring conditions. The configuration is analogous to electrical resistors in series connection. Scale bar: 40 μm. (**b**) Distinct fluidic resistances along the channel length creates a system of parallel resistors. As visualized in the *xy* micrograph, the upper half of the channel has a resistance of *r*_||_, and the lower half, *r_H_*, per unit length. Scale bar: 100 μm. (**c**) Microfluidic node with three arms, *i.e.*, a T-junction. Each arm possesses a specific value of resistance per unit length: *r*_⊥_, *r*_||_, and *r_H_*, respectively along the vertical, horizontal (right), and horizontal (left) channels. A small reservoir was placed (follow the vertical arm beyond the junction), to avoid creation of a stagnation point at the flow bifurcation. Scale bar: 100 μm. (**d**) Microfluidic node with multiple channels. The channels on the left half of the micrographs possess homeotropic anchoring, while each of the channels on right half has a specific resistance. The channel oriented parallel to the planar direction (double-headed arrow) represents the hybrid || planar case, having the minimum resistance, *r*_||_. As the angular orientation of the channel relative to the planar direction is changed, the resistance increases in the order: *r*_||_ < *r*_α_ <*r*_β_. Scale bar: 150 μm.

The discussed anisotropic attributes offer interesting avenues to tune the fluidic resistance in microchannels. For instance, spatially modulated flow fields can be created within microchannels by incorporating composite boundary conditions, as the ones shown in Figure 6. Typically, one half of the glass substrate was treated for uniform planar anchoring, and the other half for homeotropic anchoring. After a brief exposure of the PDMS relief (with the imprinted microchannel) to plasma, it was surface-bonded to the pre-treated glass substrate. The fabricated channels were left overnight, during which PDMS reverted back to its native surface properties (favouring homeotropic anchoring) [30]. The resulting anchoring on the microchannel walls is thus composite: one half homeotropic and the other hybrid || planar. As we have discussed before, microchannels possessing hybrid || planar surface anchoring has a significantly lower fluidic resistance compared to one having homeotropic anchoring. Figure 6a presents an optical micrograph (*xy* plane) of a NLC filled microfluidic device with composite anchoring conditions. As observed between crossed-polarizers, the left half of the channel is homeotropic, while the right half is functionalized to create hybrid || planar anchoring. Consequently, the fluidic resistance is higher on the left side of the channel as compared to the right side: *r_H_* > *r*_||_. In analogy to electrical circuits, this configuration presents a standard series connection of the resistors. The effective resistance per unit channel length is thus given by *r_effective_* = *r_H_* + *r*_||_. Complementing the series connection, Figure 6b shows the polarized micrograph (*xy* plane) of a fluidic circuit having a combination of resistances in parallel: The upper half of the channel has hybrid || planar anchoring, while the lower half possesses homeotropic anchoring. For an applied pressure difference, the flow through the upper part was observed to be faster than that through the lower part. The equivalent resistance per unit length is given by $\frac{1}{r_{effective}}=\frac{1}{r||}+\frac{1}{r_{H}}$. It is rather straightforward to extend the series or the parallel configuration of the fluidic resistors by repeating each unit *i.e.*, repeating the nature of the surface anchoring, alternately. Figure 6c presents a microfluidic node with three distinct anchoring conditions. Here, the three arms of the T-channel have three different fluidic resistances: *r*_⊥_, *r*_||_, and *r_H_* respectively in the lower, right, and left arms, essentially creating a fluidic junction with flow guidance capabilities. In fact, one can fabricate a range of fluidic resistances by orienting the PDMS channels appropriately on a glass surface possessing uniform planar anchoring. One such example is shown in Figure 6d. While the channels on the left half of the micrograph have all homeotropic anchoring, the ones on the right half have varying resistance. The resistance is lowest for the channel parallel to the direction of the flow (*r*_||_), and gradually goes up, *r*_α_ <*r*_β_, as the angular separation of the channel path relative to the uniform alignment on the glass surface increases. It is worthwhile to note here that the flow of an isotropic fluid does not make any distinction between these distinct regions with specific fluidic resistances. Since the channel dimensions in each case remain fixed, the surface induced variation of the nematic resistance will have no bearing on isotropic fluid flows.

**Figure 7:**
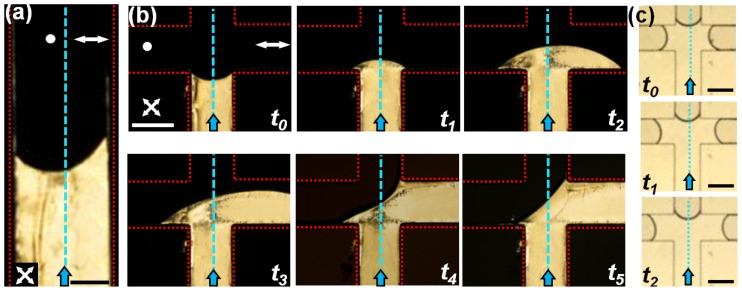
Flow guidance using T-junction microchannel. (**a**) Polarized micrograph shows the meniscus of NLC 5CB in the vertical arm of a T-junction (the crossed-arrows indicate the orientation of the polarizers). The surface anchoring is homeotropic on the left, and hybrid ⊥ planar on the right of the dotted blue line. The dotted red lines denote the channel boundaries. Note the asymmetry of the meniscus shape: the point of contact at the right wall leads the one the left wall. Scale bar: 200 μm. (**b**) Micrograph sequence demonstrating the guidance of the nematic flow at the T-junction. As time increases, the flow is guided to the right arm of the channel, which has a lower resistance, *r*_||_, relative to the left arm. Time interval between the images is not uniform. Scale bar: 400 μm. (**c**) Optical micrograph sequence showing the flow of 5CB at the T-junction in isotropic phase. No guidance was observed in this case. The volume occupied within each arm remains equal over time. Scale bar: 400 μm.

The composite anchoring templates within simple microfluidic confinements offer interesting applications including spatial modulation of flow profiles and speeds, guiding flow at a branched architecture, and separation of colloidal particles. Here we briefly demonstrate the application of an anchoring template in a T-junction as a nematofluidic valve. Using appropriate surface functionalization, the microchannel is divided symmetrically into homeotropic (left) and hybrid sections (Figure 7) about the blue dotted lines. The channel is filled through the vertical arm of the T-channel using a constant gravitational head. Interestingly, the shape of the meniscus is asymmetric in the vertical arm (Figure 7a) due to distinct anchoring conditions on either side of the channel (the red dotted lines indicate the channel walls). Figure 7b shows a time sequence of polarized optical micrographs when the flow reaches the T-junction. As seen clearly, with increasing time, the nematic flow is guided preferentially along the right arm of the channel. Over time, the flow reaches a steady state and stabilizes completely at the right arm of the microchannel (*t*_5_ of Figure 7b). Flow can be however introduced to the left arm by increasing the outlet pressure of the right arm. The subsequent flow along the homeotropic arm is preceded by disruption of the stable interface between air and nematic 5CB, aligned diagonally at the junction (see *t*_5_ of Figure 7b). The stability of this interface under different pressure conditions is currently under investigation. In contrast to the nematic case, 5CB in isotropic phase does not exhibit preferential guidance along any arm of the T-junction. This is shown in Figure 7c: both the arms of the channel have equal distribution of nematic volume at any given time.

## 3. Experimental Section

### 3.1. Materials

The experiments were carried out using single component thermotropic nematic liquid crystal 4-cyano-4-pentyl-1,1-biphenyl, commonly known as 5CB. 5CB was procured from Synthon Chemicals (Wolfen, Germany), and used without additional purification. 5CB was found to be appropriate for the experiments particularly due to the room temperature nematic phase (undergoes a transition to isotropic phase at ~35 °C), and its chemical inertness to microfluidic devices fabricated using soft lithography.

### 3.2. Microfluidic Confinement and Flow Set up

**Figure 8:**
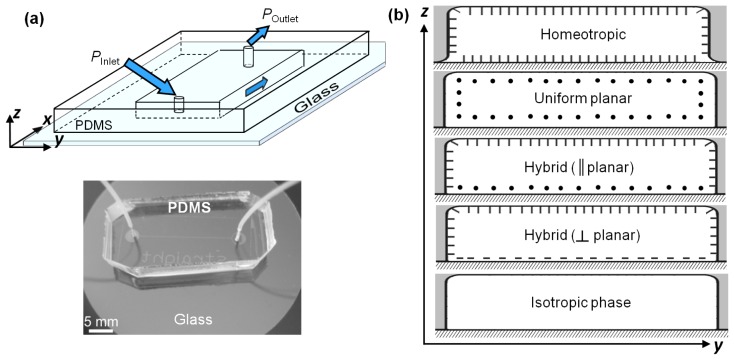
Pressure-driven nematic flow in microfluidic devices. (**a**) Schematic of the microchannel, flow is along *x* direction. The photograph below shows a typical PDMS-glass microchannel, shown here with the inlet and outlet tubing. (**b**) Orientation of the flow direction (into the image plane) relative to the surface anchoring on the channel walls, from top: homeotropic (molecules orient perpendicularly on the channel surface), uniform planar (molecules orient parallel to the channel surface, along the flow direction), hybrid || planar (molecules are perpendicular on the PDMS walls, and parallel to the glass surface, along the flow direction), hybrid ⊥ planar (molecules are perpendicular on the PDMS walls, and parallel to the glass surface, orthogonal to the flow direction), and flow in isotropic phase (~35 °C).

The nematic liquid crystal was flowed through linear microchannels having rectangular cross-sections of different dimensions. Microfluidic channels were fabricated out of polydimethylsiloxane (PDMS), a silicon-based organic polymer, on a photoresist master through a process known as soft lithography [30]. The PDMS replicas were attached to a microscope slide by exposing both surfaces to oxygen plasma (Figure 8a). The microchannels were functionalized to induce a range of nematic anchoring conditions using different physical and chemical methods. A detailed protocol for inducing appropriate boundary conditions on the microchannel walls can be found in [30] and [31]. Additional details have been discussed at appropriate sections of this paper. As summarized in Figure 8b, the primary anchoring conditions considered here include homeotropic (LC anchoring perpendicular to the surface) and uniform planar anchoring cases (LC anchoring parallel to the surface). Experiments were also carried out under hybrid boundary conditions, wherein the PDMS relief provided homeotropic anchoring, and the glass surface induced uniform planar anchoring along a specific direction relative to the flow field. Here we consider two distinct cases of hybrid anchoring: || planar: flow and the planar anchoring are parallel to each other, and ⊥ planar: flow and the planar anchoring are mutually orthogonal. At both ends of the microchannel, cylindrical holes with 750 μm diameter were punched through the PDMS to provide housing for the flow tubing (as shown in Figure 8a). Teflon tubes typically with 300 μm inner, and 760 μm outer diameters were inserted into each of the housings, and served as connectors for inlet-to-source and outlet-to-sink, respectively. Generation of a precise and controlled LC flow inside the microfluidic channels was obtained by a pressure source or a syringe pump. For pressure driven flows, the inlet was connected to a commercially available Fluigent Maesflow Flow Controller (Villejuif, France). Alternatively, pressure-driven flow was created using a gravitational head. The pressure at the channel exit was maintained at atmospheric pressure by immersing the outlet tubing within a 5CB sink placed on a sensitive mass balance, which measured the total mass of 5CB that flowed through the channel with a precision of ±100 μg. The channels could be equivalently connected to a gas-tight microliter syringe (1001LT, Hamilton Bonaduz, Bonaduz, Switzerland), and driven by a gear pump (neMESYS, Cetoni, Korbußen, Germany) to create a constant flow volume rate. The nematic flow through the microchannels was quantified by tracking tracer particles over time, or by measuring the mass of the 5CB flowing out of the channel.

### 3.3. Visualization of the Director Field and the Flow Speed

The flow-induced evolution of the nematic director, and its dynamics were studied using polarization optical microscopy (Nikon Eclipse LV100 polarization microscope, Tokyo, Japan). We have used polarized white light for the experiments using different magnifications—depending upon the region of interest and the optical resolution desired. In addition to the orthoscopic studies, conoscopy was performed by introducing a Bertrand lens between the analyzer and the ocular. The white light microscopy set up was employed for the particle tracking experiments. The tracers used in this work were typically silica particles of mean diameter ~2.5 μm. Due care was taken to avoid spontaneous aggregation of the tracers by maintaining the particle concentration very low, and using the dispersions freshly after sonication (ultra-sound bath). Using optical microscopy, movies of the nematic flow were acquired typically at a rate of 15 frames per second. The image files were then analyzed using a standard algorithm of tracking and trajectory analysis available through an open source image processing package, FIJI (formerly known as ImageJ). The low tracer density and flow speeds typically less than 1000 μm/s make the analysis straightforward, since the particles can be tracked over multiple frames separated consecutively by equal time intervals.

## 4. Conclusions

The work presented here outlines the first steps towards realization of fluidic networks by harnessing anisotropic interactions present in flows of liquid crystalline materials. Using nematic liquid crystal 5CB in microfluidic confinements, we have quantified the interactions under different boundary, and thermodynamic conditions. The results present and demonstrate accessible experimental cues to tune the fluidic resistance in microchannels by up to five times the usual hydrodynamic resistance. It is the anisotropic coupling between the flow-induced viscous and the surface-induced elastic interactions, which is fundamental in attaining this wide range of fluidic resistance in nematic microflows. For a fixed geometric dimension and flow rate (or applied pressure difference), maximum resistance was observed in microchannels possessing homeotropic anchoring, whereas the resistance was minimum when the 5CB molecules were aligned along the flow direction (uniform planar anchoring). The role of elasticity in modulating the fluidic resistance was conclusively demonstrated in homeotropic microchannels of varying depths, particularly at low flow speeds (Ericksen numbers). At a given value of the applied pressure, the fluidic resistance in the shallower channel showed ~60% enhancement over that expected from pure hydrodynamic considerations. While appropriate functionalization of the microchannels was used for passive modulation of the fluidic resistance, experiments conducted at elevated temperatures indicate interesting possibilities for active control of the flow properties. Experimentally, we induced a transition of the nematic 5CB to isotropic phase, thereby reducing the resistance by a half in channels with uniform planar anchoring, and by a third in homeotropic microchannels. The combination of active and passive modulation cues provides a broad parameter space to design microfluidic networks of hierarchical complexity. As a simple demonstration, we have presented microchannels with different combination of fluidic resistances: series, parallel, and nodes with three or more distinct values of resistance. The three element node was applied to create a simple nematofluidic valve. Integrating optical techniques to create precise anchoring motifs within the microchannels warrant interesting possibilities in templating flow profiles. In addition to other potential applications including tunable flow profiles, flow and colloidal guidance, and microscale mixers, the concepts presented here trigger some open questions, fundamental to the understanding of complex flow patterns. For instance, efforts are currently underway to understand the morphology of the liquid crystal meniscus under composite boundary conditions, and its role in capillary flow dynamics. Furthermore, introduction of an external temperature or electric field promises interesting competitive effects in the physics of anisotropic fluid flows.
